# Correction to ‘Cell Membrane Vesicles Encapsulating Il24 mRNA With Enriched CD6 Display Exhibit Enhanced Targeted Antitumor Efficacy’

**DOI:** 10.1002/jev2.70327

**Published:** 2026-06-17

Wang, D., R. Gao, D. Liu, et al. 2026. “Cell Membrane Vesicles Encapsulating Il24 mRNA With Enriched CD6 Display Exhibit Enhanced Targeted Antitumor Efficacy.” Journal of Extracellular Vesicles15, no. 6: e70299. https://doi.org/10.1002/jev2.70299.

In the originally published article, author Bin Xia was not included as a corresponding author. The corresponding author list should have appeared as shown below. The online version of the article has been updated.

Correspondence: Shengjie Jiang (kqjiangshengjie@bjmu.edu.cn), Bin Xia (xiabin@pkuss.bjmu.edu.cn)

We apologize for this error.

